# Intra-atrial Approach for Infracardiac Total Anomalous Pulmonary Venous Connection

**DOI:** 10.1016/j.atssr.2022.07.001

**Published:** 2022-07-21

**Authors:** Makoto Nakamura, Kazuyoshi Kanno, Masahiko Nishioka

**Affiliations:** 1 Department of Pediatric Cardiovascular Surgery, Okinawa Prefectural Nanbu Medical Center & Children’s Medical Center, Okinawa, Japan

## Abstract

Patients with total anomalous pulmonary venous connection have various forms of pulmonary veins. Because pulmonary vein obstruction is a major and critical complication, it is necessary to create as large an anastomosis as possible while considering the positional relationship with the left atrium. Here, we report the case of a female neonate diagnosed with infracardiac total anomalous pulmonary venous connection and hypoplastic common pulmonary vein. The operation was performed by a sutureless technique in the intra-atrium. The patient was discharged from the hospital 20 days after operation with no complications.

Regarding the surgical strategies of total anomalous pulmonary venous connection (TAPVC) repair, the sutureless technique is a common technique to avoid postoperative pulmonary vein (PV) obstruction. The approach is chosen after considering the morphologic features of the individual PV structures. Here, we report good results obtained by performing surgery with the intracardiac approach in a patient with tree-shaped infracardiac TAPVC.

A female neonate weighing 3.1 kg and born at 40 weeks of gestation was transferred to our hospital because of hypoxia (postnatal saturation in oxygen, 80%). Imaging findings led to a diagnosis of infracardiac TAPVC. Computed tomography showed that her common PV was hypoplastic; her right PV merged into one PV in the lung and formed a long common branch; and her left PV had upper and lower portions, which became a short common branch behind the inferior vena cava–right atrium junction (Figure 1). She did not have severe PV obstruction; however, she had a small left atrium, small left ventricular chamber, and reverse flow over the right brachial artery in the descending aorta. After improvement in antegrade blood flow was confirmed at 7 days of age, operation was performed.

**Figure 1:**
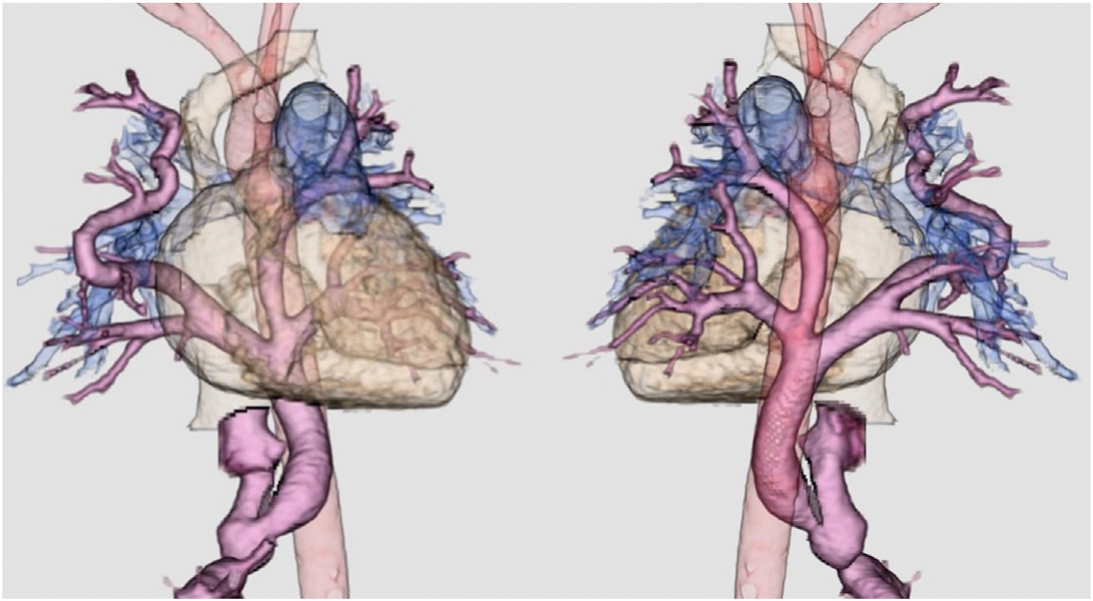
Computed tomography findings. The common pulmonary vein (PV) is hypoplastic. The right PV merges into 1 PV in the lung and forms a long branch, and the left PV has upper and lower portions, which become a short common branch behind the inferior vena cava–right atrium junction.

Cardiopulmonary bypass was initiated after ascending aortic and bicaval cannulation. After cardiac arrest, the right atrium was incised, and the primary atrial septal membrane was resected. The descending vein was ligated, and the left upper PV was incised above the pericardium. An incision was made in each PV branch and descending vein to secure a 10-mm opening (Figure 2A). The right atrial incision was extended to the atrial septum, and the left atrial wall was incised widely to the base of the left atrial appendage. The pericardium at the margin of the PV incision and left atrium were sutured from the intra-atrial side. In addition, the right atrial wall was sutured to the right side of the right PV incision to complete the PV–left atrium anastomosis (Figure 2B). The atrial septal defect was closed with a pericardial patch (Figure 2C). After the cross-clamp was released, cardiopulmonary withdrawal was attempted. However, she was returned to the intensive care unit on venoarterial extracorporeal membrane oxygenation because of ventilatory insufficiency from pulmonary hemorrhage. The operation time was 4 hours 43 minutes, the cardiopulmonary bypass time was 3 hours 10 minutes, and the cross-clamp time was 1 hour 8 minutes. Extracorporeal membrane oxygenation was withdrawn on the operative day, and a 2-stage chest closure was performed on the fifth postoperative day. Echocardiographic examination at discharge revealed an anastomotic area of 5.3 × 6.2 mm and a pressure gradient of 2 mm Hg with no stenosis. The patient was discharged 20 days after the surgery without complications. Two months after the operation, no complications were found on echocardiography, and catheterization will be performed regularly to evaluate the PVs.

**Figure 2:**
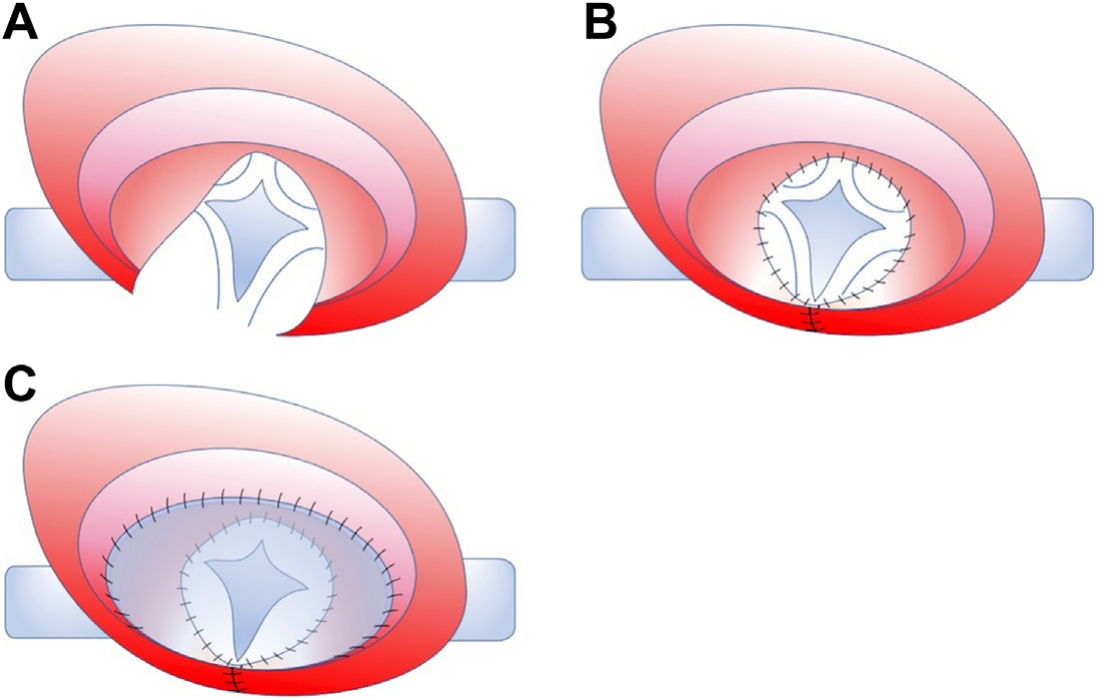
Schema of the surgical procedure. (A) An incision is made in each pulmonary vein (PV) branch and descending vein to secure a 10-mm opening. The right atrial incision is extended to the atrial septum, and the left atrial wall is incised wide to the base of the left atrial appendage. (B) The pericardium at the margin of the PV incision and left atrium are sutured from the intra-atrial side. The right atrial wall is sutured on the right side of the right PV incision to complete the PV–left atrium anastomosis. (C) The atrial septal defect is closed with a pericardial patch.

## Comment

Although the proportion of pulmonary venous stenosis has decreased since the sutureless method was introduced in the treatment of TAPVC, it is still reported to occur at a rate of 0% to 8% only.^1^, ^2^, ^3^ In addition, heterotaxy syndrome, single ventricle, and mixed-pattern TAPVC are thought to have a higher frequency of postoperative PV stenosis.^4^ The tree-shaped infracardiac TAPVC is also thought to belong to these groups; our patient had the same PV morphologic appearance.

Kawashima and colleagues^5^ have reported detailed treatment of tree-shaped infracardiac TAPVC. Like their patient, our patient had almost no common PV. To obtain a large anastomotic opening, the branch of the PV was cut. Goor and coworkers^6^ reported an intrapericardial approach for the supracardiac/cardiac-type TAPVC with a small left atrium. We used a similar approach to obtain a wide left atrial anastomosis. Their approach made it possible to suture the pericardium around the venous anastomosis opening in the same field of view as the incision site in multiple directions.

The intra-atrial approach is not necessary to expand the atrium or ventricle from the outside at the time of anastomosis and is considered to avoid excessive tension or loosening at the anastomotic site. This approach for infracardiac TAPVC has been reported only a few times; however, this surgical procedure may be useful in certain patients.
